# Which Urban Migrants Default from Tuberculosis Treatment in Shanghai, China?

**DOI:** 10.1371/journal.pone.0081351

**Published:** 2013-11-28

**Authors:** Jing Chen, Lihong Qi, Zhen Xia, Mei Shen, Xin Shen, Jian Mei, Kathryn DeRiemer, Zheng’an Yuan

**Affiliations:** 1 Department of Tuberculosis Control, Shanghai Municipal Center for Disease Control and Prevention, Shanghai, China; 2 Department of Public Health Sciences, University of California Davis, School of Medicine, Davis, California, United States of America; Institut de Pharmacologie et de Biologie Structurale, France

## Abstract

**Background:**

Migration is a major challenge to tuberculosis (TB) control worldwide. TB treatment requires multiple drugs for at least six months. Some TB patients default before completing their treatment regimen, which can lead to ongoing infectiousness and drug resistance.

**Methods:**

We conducted a retrospective analysis of 29,943 active TB cases among urban migrants that were reported between 2000 to 2008 in Shanghai, China. We used logistic regression models to identify factors independently associated with treatment defaults in TB patients among urban migrants during 2005-2008.

**Results:**

Fifty-two percent of the total TB patients reported in Shanghai during the study period were among urban migrants. Three factors increased the odds of a treatment default: case management using self-administered therapy (OR, 5.84, 95% CI, 3.14-10.86, p<0.0005), being a retreatment case (OR, 1.47, 95% CI, 1.25-1.71, p<0.0005), and age >60 years old (OR, 1.33, 95% CI, 1.05-1.67, p=0.017). The presence of a cavity in the initial chest radiograph decreased the odds for a treatment default (OR, 0.87, 95% CI, 0.77-0.97, p=0.015), as did migration from central China (OR, 0.85, 95% CI, 0.73-0.99, p=0.042), case management by family members (OR, 0.73, 95% CI 0.66-0.81, p<0.0005), and the combination of case detection by a required physical exam and case management by health care staff (OR, 0.64, 95% CI, 0.45-0.93, p=0.019).

**Conclusion:**

Among TB patients who were urban migrants in Shanghai, case management using self-administered therapy was the strongest modifiable risk factor that was independently associated with treatment defaults. Interventions that target retreated TB cases could also reduce treatment defaults among urban migrants. Health departments should develop effective measures to prevent treatment defaults among urban migrants, to ensure completion of therapy among urban migrants who move between cities and provinces, and to improve reporting of treatment outcomes.

## Introduction

Tuberculosis (TB) remains one of the most significant infectious diseases worldwide. In 2011, there were an estimated 8.7 million incident TB cases (i.e., 125 cases per 100,000 population) globally, and China had over one million incident TB cases (i.e., 75 cases per 100,000 population), the second largest number in the world [1]. 

Migration is one of the major challenges to TB control in both developed and developing countries. In some countries with a low incidence of TB, the majority of TB cases were among immigrants who arrived from other countries [2,3]. However, migration within a country is also common, particularly migration from rural to urban areas. Migrants can have an increased risk of developing and transmitting diseases due to their limited access to housing, education and health care services [4] and the economic hardships of illness and treatment [5]. Previous studies showed that migrant TB patients often lose their jobs and face financial hardships during their treatment [6], resulting in poor treatment outcomes. TB patients who interrupt or default from treatment may remain infectious and may develop multidrug-resistant (MDR) TB, increasing the public health problem that is TB in China. 

With the rapid socioeconomic development that is occurring in China, an increasing number of rural residents move to urban areas and become urban migrants within China. There were over 145 million urban migrants in China in 2009, a number greater than the total population size of many countries. Over 75% of the urban migrants in China have only a primary education, most work in manufacturing, construction and service industries[7], and many live and work in conditions that promote TB transmission [8,9].

Shanghai is one of the most developed metropolitan areas in China. The notification rate of pulmonary TB among the13.8 million local residents in Shanghai was 39.4/100,000 population in 2000 [10,11], lower than the national rate of 41.7/100,000 population in the same year [12]. However, the urban migrant population increased from 3.9 million in 2000 to 6.6 million in 2007 [13], creating challenges for TB control in Shanghai. 

Urban migrants may have cultures and socioeconomic backgrounds that are different from the local residents, and the risk factors for a treatment default among urban migrants in Shanghai may be different from those previously reported in other countries. The objectives of this study were: 1) to describe the characteristics of TB patients among the urban migrant population during 2000-2008 in Shanghai, China; and 2) to identify the risk factors for defaulting from anti-TB treatment among urban migrants in Shanghai during 2005-2008. We were particularly interested in risk factors that can be modified to reduce treatment defaults, improve treatment outcomes, and make an impact on the overall TB epidemic in Shanghai, China.

## Materials and Methods

### Study Area

Shanghai is one of the four provincial-level municipalities in China and has 18 administrative districts. The population of Shanghai was approximately 19.2 million in 2009, including 13.8 million local residents and an estimated 5.4 million urban migrants. The Shanghai Municipal Center for Disease Control and Prevention (CDC) manages the Tuberculosis Control Program for the municipality of Shanghai. 

### TB Control Network and Program

Shanghai has an integrated TB control network involving CDCs, TB hospitals, general hospitals and community health centers (CHCs). CDCs are mainly responsible for epidemiological surveillance and for developing and implementing TB control programs and strategies. TB hospitals are mainly responsible for TB case diagnosis, treatment, and case notifications. General hospitals perform case detection and refer TB suspects to TB hospitals. CHCs are responsible for finding and tracking the suspect TB cases that are referred to the TB hospitals by the general hospitals, and for performing case management and health education [11].

A complete surveillance system and mandatory reporting system were established in Shanghai in the 1990s. Shanghai CDC recorded demographic, clinical and mycobacteriological information on every individual diagnosed with pulmonary TB. The TB control program in Shanghai is financially supported by China’s Central government and the Shanghai municipal government. In 2004, an enhanced TB control program was implemented in Shanghai, offering free TB-related screening and diagnosis to TB suspects, as well as free anti–TB treatment to diagnosed TB cases, among both local residents and urban migrants. However, for TB cases with severe TB or other comorbidities, the TB patients were often hospitalized and needed to pay for any additional examinations and treatment. 

### Study Population

A TB patient is defined as an individual who was sputum smear positive or culture positive for *Mycobacterium tuberculosis*, or sputum smear or culture negative for *M. tuberculosis* but with other signs and symptoms compatible with TB (e.g., abnormal chest radiograph, abnormal chest computerized tomography scan, or clinical evidence of active disease). An urban migrant is defined as an individual who left his/her hometown where he/she was registered after birth, and stayed in Shanghai without obtaining local permanent residency in Shanghai. A treatment defaulter is defined as a patient who took anti-TB treatment intermittently over any two-month period, or a patient who interrupted their treatment without follow-up by TB control staff and without additional information [14]. A retreated TB case was defined as a patient who previously received at least one month of anti-TB therapy. 

We used the information available for all pulmonary TB patients registered in the Shanghai CDC surveillance system among the urban migrants during 2000-2008. The information collected for each TB case includes age, sex, town of permanent residence, occupation, history of previous anti-TB treatment, case detection method, initial chest radiograph results, mycobacteriological test results, method of case management, and treatment outcome. Occupation was categorized as light manual workers (including waiters, babysitters, or workers with other light manual jobs which were likely to be indoors and did not require much physical work), heavy manual workers (including construction workers, farmers, taxi and bus drivers), and other workers (including health workers, teachers, students, retired persons, pre-school children and those who did not state their occupations). The town of permanent residence was used to create categories by foreign country and region of China (north, south, east, west). Pulmonary TB patients were classified as new cases if they did not previously ever take anti-TB drugs for more than one month. Retreated cases were TB patients who previously received treatment for at least one month, were previously cured, and were once again sputum smear positive. Cases were detected by at least one of three methods: 1) the patient actively sought health care, 2) health examinations were required by their current or future employers, or 3) contact investigations were conducted by the public health department. The covariates created above were used in the analyses.

**Figure 1:**
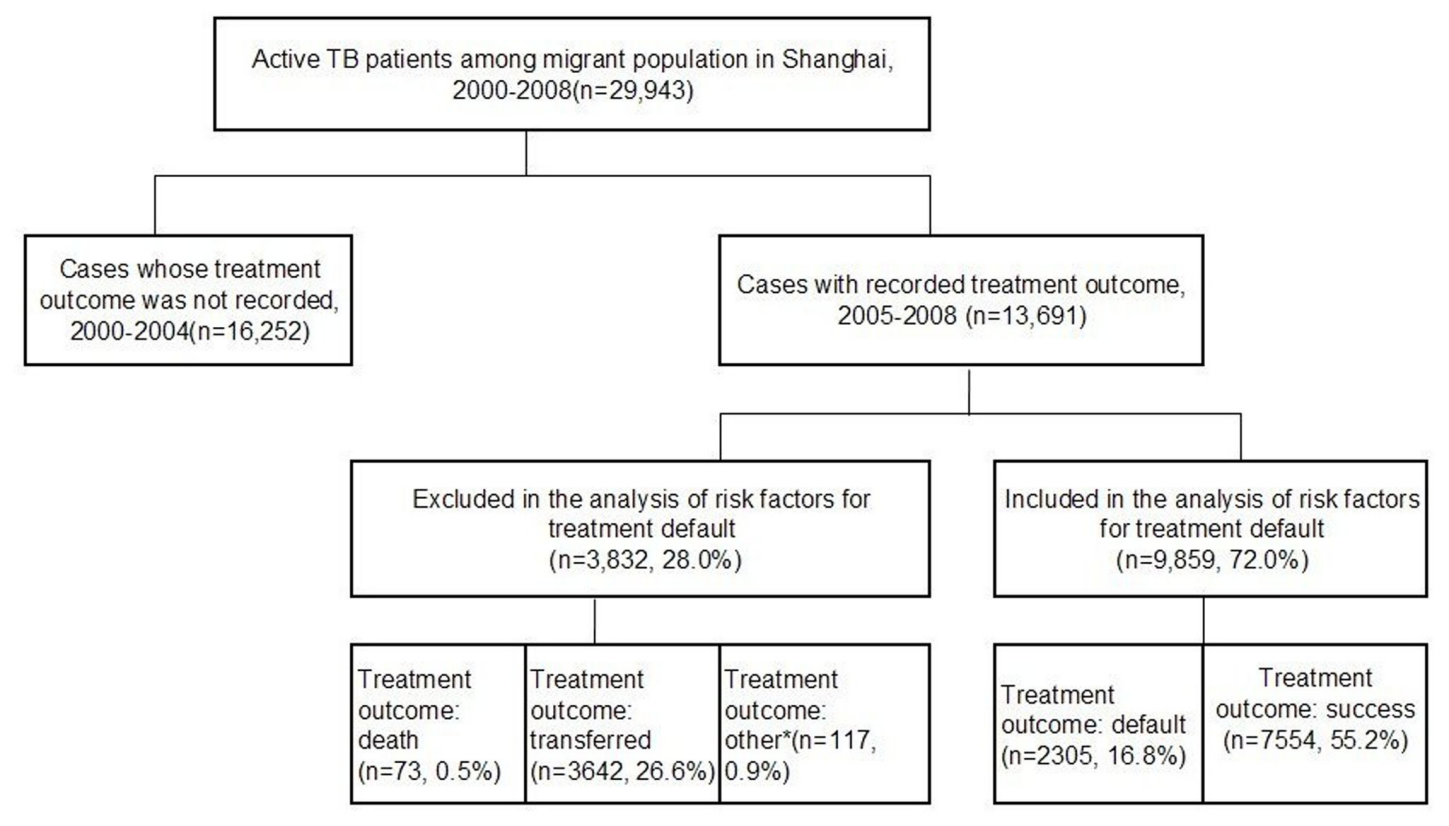
Study population of active tuberculosis (TB) patients among urban migrants in Shanghai, 2000-2008. Cases with treatment outcomes other than treatment success or treatment default were excluded from the analyses. *Includes patients whose tests indicated they did not have tuberculosis and whose anti-TB therapy was therefore discontinued, and TB patients who were still on treatment after one year of therapy.

Information about treatment outcomes and patient management among migrant TB patients was recorded and verified beginning in 2005. Therefore, we analyzed only the data from TB cases among urban migrants reported during 2005-2008 to identify the risk factors associated with a treatment default. We excluded cases with treatment outcomes other than treatment success or treatment default, such as transferring out of the municipality, death with TB, or other outcomes (Figure 1). 

### Statistical Analysis

We calculated the frequencies and the notification rates of active TB cases and sputum smear positive TB patients among urban migrants in Shanghai during 2000-2008. The notification rate was calculated based on migrant population data provided by the Shanghai Municipal Statistic Bureau in 2000, 2003 and from 2005 to 2008, when data were available [10]. The numerator was the number of the newly-registered active or sputum smear positive TB patients and the denominator was the estimated urban migrant population in the corresponding year. 

We divided the study into two periods: 2000-2004 and 2005-2008. In 2004, the Shanghai government started to implement an enhanced TB control program, providing free TB-related screening, diagnosis and treatment to suspected and confirmed TB patients. For each covariate, we obtained the frequencies and proportions of TB patients among urban migrants for each covariate between the two periods using the chi-squared (*χ* ^2^) test. We also examined treatment outcomes and conducted the chi-squared test for linear trend of treatment defaults during 2005-2008.

We used simple and multiple logistic regression models to investigate whether there were significant associations between each of the potential risk factors and the outcome, treatment default (yes or no), and to obtain odds ratios (OR) and 95% confidence intervals (CI). We considered interactions between two factors, when appropriate. We used a forward stepwise method to build the multiple logistic regression models, and the likelihood-ratio test to compare models. All hypothesis tests were two-sided and a P value ≤0.05 was considered significant. All analyses were conducted using Stata statistical software (release 11, Stata Corporation, College Station, Texas, USA). 

### Ethical considerations

The Ethical Review Committee at Shanghai Municipal Center for Disease Control and Prevention approved this study. The ethics committees waived the need for patient consent because it was a retrospective study, and patients’ personal identifiers were not disclosed and were not used during the entire study.

## Results

### Characteristics of TB Cases Among Urban Migrants

**Figure 2:**
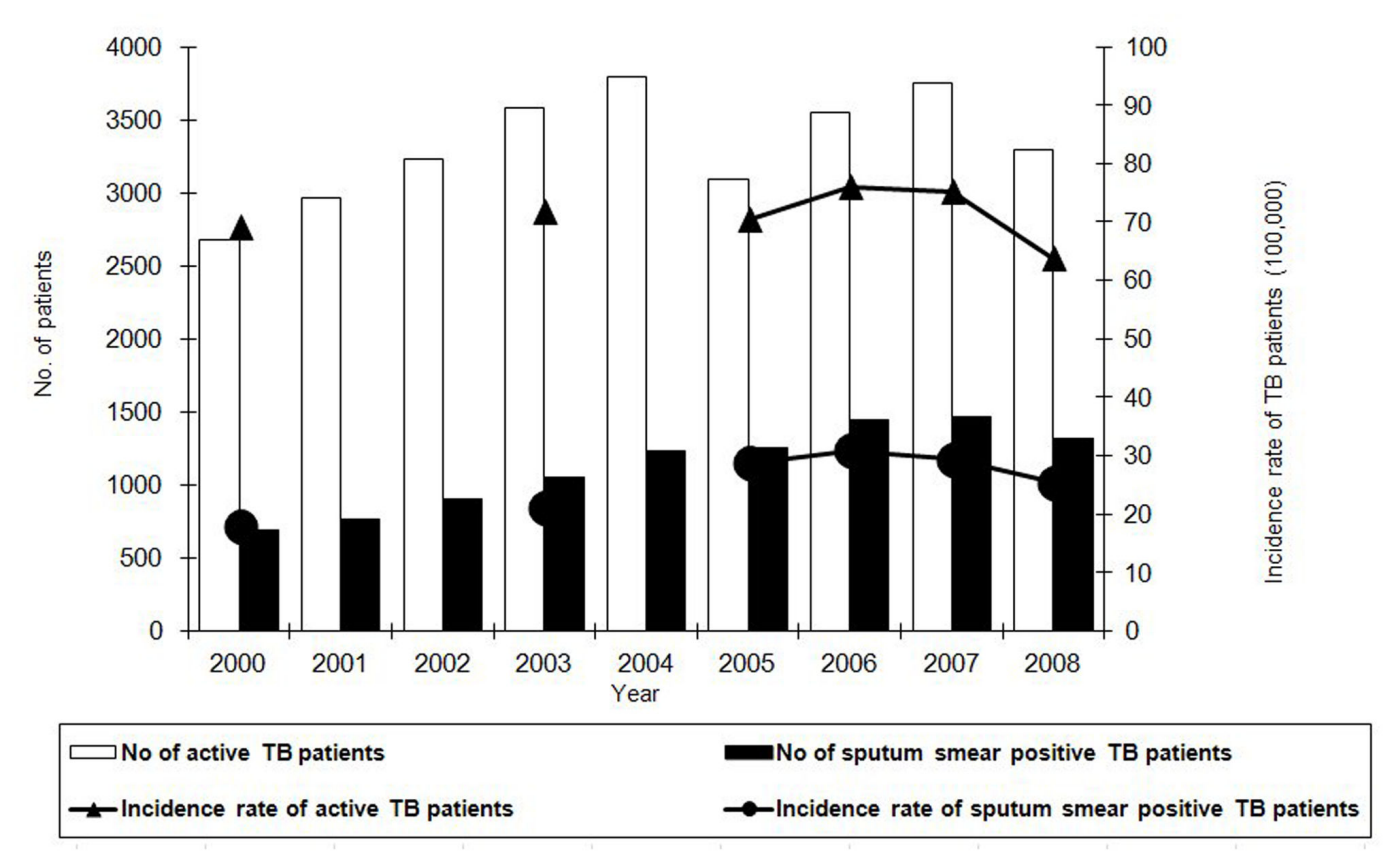
Tuberculosis case numbers and notification rates among urban migrants in Shanghai, 2000-2008. The notification rates varied from 76.7/100,000 population in 2000 to 75.2/100,000 population in 2007. Sputum smear positive patients (n=10,118) accounted for one third of the all patients. The overall notification rate of sputum smear positive TB patients among urban migrants was 25.7/100,000 population and fluctuated each year.

There were 29,943 active TB cases among urban migrants in Shanghai reported during 2000-2008, accounting for 55.2% of the total TB cases (Shanghai CDC, unpublished data). The overall notification rate of active TB patients among urban migrants was 71.1/100,000 population. The notification rates varied from 76.7/100,000 population in 2000 to 75.2/100,000 population in 2007, but significantly decreased to 63.8/100,000 population in 2008 (P <0.0005). Approximately one third of the patients (n=10,118) were sputum smear positive. The overall notification rate of sputum smear positive TB patients among urban migrants was 25.7/100,000 population, which increased from 19.7/100,000 population in 2000 to 30.8/100,000 population in 2006, then decreased to 25.4/100,000 population in 2008 (Figure 2). 

We compared the characteristics of the study subjects between the two study periods (Table 1). Notably, the proportion of TB patients who were female, age 15–29 years old at TB diagnosis, migrated from western, northern or central China, were light manual workers or other workers, and retreated cases significantly increased from 2000-2004 to 2005-2008. The proportions of TB patients among urban migrants who had a sputum smear test performed and/or a sputum culture performed and who were diagnosed because they sought health care services, also significantly increased by 2005-2008. 

**Table 1:** Characteristics of Tuberculosis Patients Among Urban Migrants in Shanghai, 2000-2004 versus 2005-2008.

	2000-2004		2005-2008		
	(n=16,252)		(n=13,691)		
Characteristics	No.	%	No.	%	*P**^a^***
Sex					<0.0005
Male	10743	66.1	8653	63.2	
Female	5509	33.9	5038	36.8	
Age, years					<0.0005
0-14	248	1.5	139	1.0	
15-29	6865	42.2	6414	46.9	
30-44	5708	35.1	4761	34.8	
45-59	2156	13.3	1577	11.5	
>60	1275	7.9	800	5.8	
Permanent area of residence in China					<0.0005
East	10458	64.4	6901	50.4	
West	2629	16.2	3304	24.1	
South	851	5.2	660	4.8	
North	737	4.5	821	6.0	
Central	1460	9.0	1729	12.6	
Foreign	37	0.2	37	0.3	
Unknown	80	0.5	239	1.8	
Occupation					<0.0005
Heavy manual workers	8190	50.4	5243	38.3	
Light manual workers	4001	24.6	4491	32.8	
Others**^*b*^**	2655	16.3	2763	20.2	
Unknown	1406	8.7	1194	8.7	
History of prior anti-TB treatment					<0.0005
New case	15042	92.6	12092	88.3	
Retreated case	1210	7.4	1599	11.7	
Cavity in the initial chest radiograph					<0.0005
Yes	5254	32.3	3203	23.4	
No	10792	66.4	10275	75.0	
Suspected cavity	206	1.3	213	1.6	
Sputum smear test					<0.0005
Done	11791	72.6	13325	97.3	
Not done	4461	27.4	366	2.7	
Sputum culture test					<0.0005
Done	586	3.6	8585	62.7	
Not done	15666	96.4	5106	37.3	
Case detection method					<0.0005
Sought health care services	14047	86.4	12290	89.8	
Required physical exam	550	3.4	1385	10.1	
Other	18	0.1	15	0.1	
Missing	1637	10.1	1	0.0	

^***a***^ Based on *χ* ^2^test of proportions.

^***b***^ Included health workers, teachers, students, pre-school children and retired persons.

**Figure 3:**
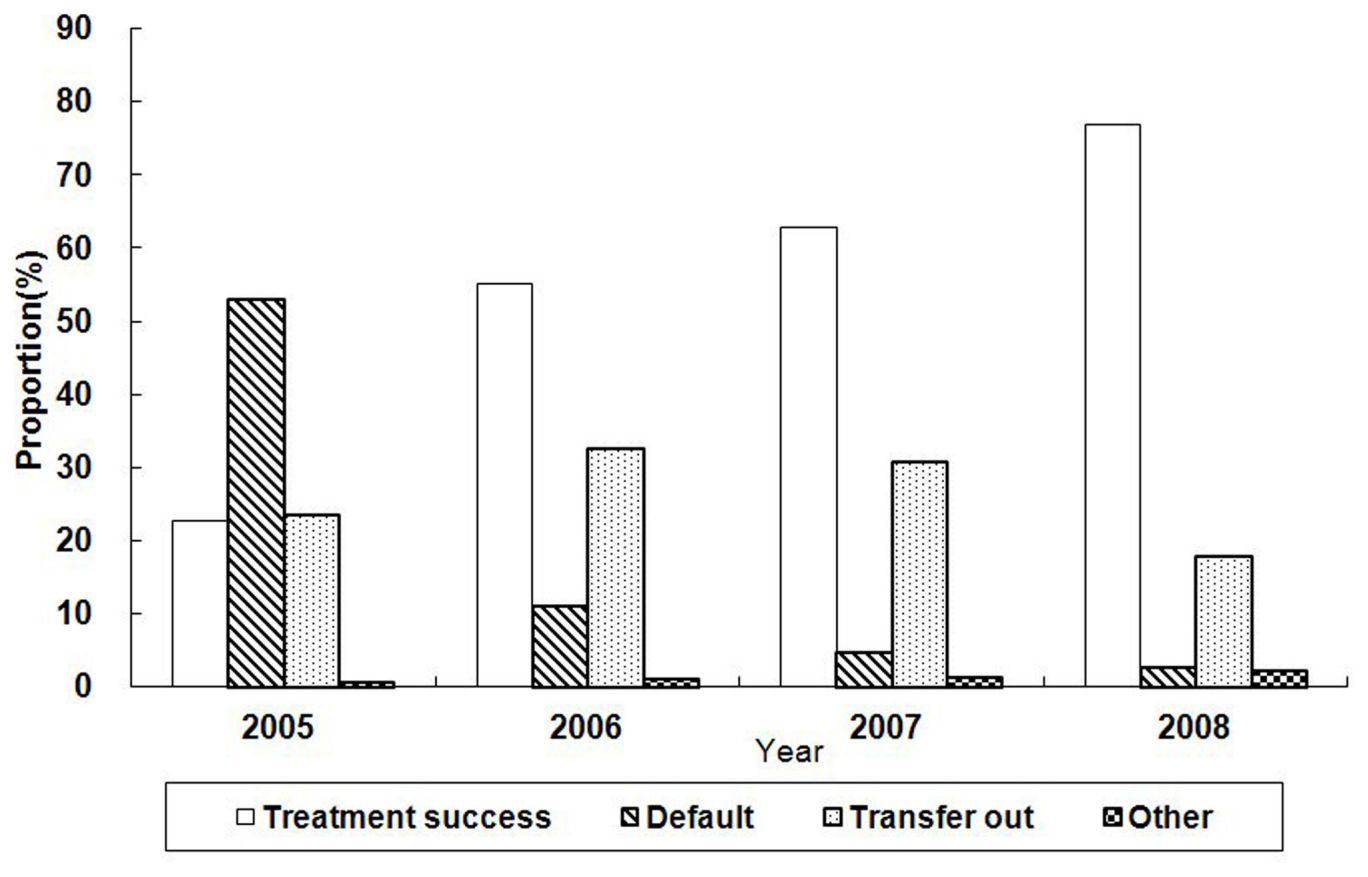
Treatment outcomes of active TB patients among urban migrants in Shanghai, 2005-2008. The average treatment success and default rates during 2005 to 2008 were 55.2% and 16.8%, respectively. There was an increasing trend for treatment success from 2005 to 2008.

The treatment outcomes of TB patient among urban migrants who were reported during 2005-2008 are presented in Figure 3. The overall treatment success and default rates for this period were 55.2% and 16.8%, respectively. The treatment success rate increased significantly from 22.7% in 2005 to 76.9% in 2008 (P <0.0005) while the treatment default rate decreased significantly from 53.1% in 2005 to 2.8% in 2008 (p<0.0005). Of the 13,691 TB cases among urban migrants during 2005-2008, 92 (0.7%) had case management using self-administered therapy while 6,216 (45.4%) TB cases were managed by family members and 7166 (52.3%) TB cases were managed by health care workers during their anti-TB treatment. 

### Risk factors for defaulting from treatment, 2005-2008

We identified the characteristics of the TB patients among urban migrants in Shanghai from 2005 to 2008 who had a treatment default versus treatment success (Table 2). We used simple logistic regression models to identify potential risk factors for defaulting from treatment. The following factors were significantly associated with defaulting from treatment (all p<0.005): age >60 years old, retreatment for TB, positive sputum smear result, and self-administered therapy. Factors that were negatively associated with treatment default were case detection by a required physical exam and case management by family members (Table 3). 

**Table 2:** Characteristics of Tuberculosis Patients with Treatment Default and Treatment Success Among Migrant Tuberculosis Patients, Shanghai, 2005–2008.

Characteristics	Treatment Default		Treatment Success		
	(n=2305)		(n=7554)		
	No.	%	No.	%	
Sex					
Female	857	37.2	2838	37.6	
Male	1448	62.8	4716	62.4	
Age, years					
0-14	25	1.1	52	0.7	
15-29	1085	47.1	3741	49.5	
30-44	816	35.4	2753	36.4	
45-59	250	10.8	723	9.6	
>60	129	5.6	285	3.8	
Permanent residency area in China**^*a*^**					
South	116	5.1	366	4.9	
West	558	24.7	1987	26.8	
East	1141	50.5	3539	47.8	
North	168	7.4	466	6.3	
Central	276	12.2	1053	14.2	
Occupation**^*b*^**					
Heavy manual workers	900	41.6	2899	42.4	
Light manual workers	810	37.4	2474	36.1	
Others**^*c*^**	454	21.0	1474	21.5	
History of prior anti-TB treatment					
New case	2015	87.4	6909		91.5
Retreated case	290	12.6	645		8.5
Case detection methods**^*d*^**					
Seeking health services	2096	91.1	6600		87.4
Required physical exam	205	8.9	948		12.6
Cavity in the initial chest radiograph**^*e*^**					
Yes	1748	77.3	5662		76.1
No	514	22.7	1776		23.9
Sputum smear test result**^*f*^**					
Negative	1332	58.5	4585		61.9
Positive	943	41.5	2828		38.1
Case management method**^*g*^**					
Self-administered therapy	32	1.4	15		0.2
Managed by health staff	1203	52.6	3378		44.9
Managed by families	1050	46.0	4128		54.9

^***a***^ Excluded the patients from foreign countries and missing data.

^***b***^ Excluded the patients with an unclear occupation.

^***c***^ Included health workers, teachers, students, pre-school children and retired persons.

^***d***^ Excluded the patients who were detected by other methods.

^***e***^ Excluded patients with a suspected cavity in the initial chest radiograph.

^***f***^ Excluded patients with contaminated or missing sputum smear test results.

^***g***^ Excluded patients with missing information for the type of case management.

**Table 3:** Unadjusted Odds Ratios for Risk Factors Associated with Treatment Default in Tuberculosis Patients Among Urban Migrants, Shanghai, 2005–2008.

Characteristics	Odds ratio (OR)	(95% CI)	*P*
Age, years			
0-14	1.70	(1.02, 2.68)	0.040
15-29	1.00		
45-59	1.19	(1.02, 1.40)	0.030
>60	1.56	(1.25, 1.94)	<0.0005
Permanent residency area in China**^*a*^**			
West	0.87	(0.78, 0.98)	0.019
East	1.00		
Central	0.81	(0.70, 0.94)	0.006
History of previous anti-TB treatment			
New case	1.00		
Retreated case	1.54	(1.33, 1.79)	<0.0005
Case detection methods**^*b*^**			
Seeking health services	1.00		
Required physical exam	0.68	(0.58, 0.80)	<0.0005
Sputum smear test result**^*c*^**			
Negative	1.00		
Positive	1.15	(1.04, 1.26)	0.005
Case management method**^*d*^**			
Self-administered therapy	5.99	(3.23, 11.10)	<0.0005
Managed by health care staff	1.00		
Managed by families	0.71	(0.65, 0.79)	<0.0005

^***a***^ Excluded the patients from foreign countries.

^***b***^ Included health workers, teachers, students, pre-school children and retired persons.

^***c***^ Excluded the patients who were detected by other methods.

^***d***^ Excluded patients with missing information for the type of case management.

We developed a multiple logistic regression model of the factors associated with treatment defaults among urban migrants (Table 4). Compared to case management by health care staff, case management using self-administered therapy significantly increased the odds for a treatment default (OR, 5.84, 95% CI, 3.14-10.86) and case management by family members significantly decreased the odds for a treatment default (OR, 0.73, 95% CI, 0.66-0.81). The odds of a treatment default also increased among urban migrants who were retreated TB patients (OR, 1.47, 95% CI, 1.25-1.71) and TB patients >60 years old (OR, 1.33, 95% CI, 1.05-1.67). The presence of a cavity in the initial chest radiograph decreased the odds for a treatment default (OR, 0.87, 95% CI, 0.77-0.97, p=0.015), as did migration from central China (OR, 0.85, 95% CI, 0.73-0.99,p=0.042), case management by family members (OR, 0.73, 95% CI 0.65-0.91, p<0.0005), and the combination of case detection by a required physical exam and case management by health care staff (OR, 0.64, 95% CI, 0.45-0.93, p=0.019).

**Table 4:** Adjusted Odds Ratios for Risk Factors Associated with Treatment Default in Tuberculosis Patients Among Urban Migrants, Shanghai, 2005–2008.

Characteristics	Adjusted OR	(95% CI)	*P*
Age (years)			
15-29	1.00		
>60	1.33	(1.05, 1.67)	0.017
Permanent residency area in China**^*a*^**			
East	1.00		
Central	0.85	(0.73, 0.99)	0.042
History of previous anti-TB treatment			
New	1.00		
Retreated	1.47	(1.25, 1.71)	<0.0005
Cavity in the initial chest radiograph**^*b*^**			
No	1.00		
Yes	0.87	(0.77, 0.97)	0.015
Type of case management			
Self-administered therapy	5.84	(3.14, 10.86)	<0.0005
Managed by health staff	1.00		
Managed by families	0.73	(0.66, 0.81)	<0.0005
Case detection by physical exam	0.64	(0.45, 0.93)	0.019
* patient managed by health care staff**^*c*^**			

^***a***^ Excluded the patients from foreign countries.

^***b***^ Excluded patients with a suspected cavity in the initial chest radiograph.

^***c***^ Interaction term is (TB patients detected by required physical exam)X (treatment was managed by health care staff).

## Discussion

Our study of TB patients among urban migrants during 2000-2008 in Shanghai, China, has several interesting findings. First, urban migrants accounted for more than half the TB burden in Shanghai. Second, although the enhanced TB control program improved the urban migrants’ access to health care services in Shanghai and significantly increased the number of patients with a bacteriologically confirmed diagnosis of TB during 2005-2008, the treatment success rate among TB patients who were urban migrants in Shanghai was below the target of 85% set by the World Health Organization (WHO). Many urban migrant TB patients transferred out of Shanghai during their treatment, and the Shanghai TB control program did not learn whether they completed therapy. Third, the type of case management during therapy was the strongest modifiable risk factor independently associated with the treatment outcome. Urban migrants with TB under self-administered therapy were more likely to default from treatment than urban migrants with TB whose therapy was administered by health care staff. Patients whose TB treatment was managed by family members also had a reduced odds of a treatment default. Finally, retreated patients were more likely to default from treatment. 

The present study has the advantage of using a comprehensive surveillance system and a large sample size over a nine-year study period in a large metropolitan area that is the destination of many urban migrants in China. Our findings can inform health departments and help them develop effective measures to prevent treatment defaults, and thus have a very practical role for TB control and prevention in China. 

Our study has several weaknesses due to its retrospective nature. Drug resistance can be a significant risk factor for a defaulting from treatment [15], but drug susceptibility test results were lacking for many TB patients in our study. Urban migrants were less likely than local residents to have steady jobs, and were more likely to leave Shanghai after getting a TB diagnosis [6]. Therefore, our study may underestimate the measure of the association between unemployment and defaulting from TB treatment. Other potential risk factors for defaulting from treatment, such as adverse side effects of the anti-TB medications [16] and lack of social support and incentives to complete anti-TB therapy [17], were not evaluated in the present study and should be considered in future research. 

Some previous studies determined that unemployment was significantly associated with treatment defaults [17,18], whereas we did not. A randomized controlled trial in Thailand showed a higher treatment completion rate among patients under directly observed therapy (DOT) by health workers, community members and family members than among patients using self-administered therapy [19]. However, a randomized controlled trial in Pakistan reported no significant differences in cure rates and treatment completion rates between the three different case management strategies [20]. Further study is needed to obtain the best treatment outcomes for the large numbers TB cases among urban migrants in Shanghai.

Our findings have implications for policies that can improve TB control in China. Collaborations between health departments in Shanghai and other geographical areas are needed to ensure that TB patients who transfer out of Shanghai successfully complete an appropriate regimen of anti-TB therapy and their treatment outcome is reported. Other large cities in China face similar challenges; between 1997 and 2002 in Beijing, the capital city of China, only 50.0% of urban migrants with TB were successfully treated, and 38.5% transferred out of Beijing and their final treatment outcome was unknown [21]. The WHO guidelines for TB treatment indicate that any willing person acceptable to the patient and answerable to the health care system can provide directly observed therapy and treatment support [22]. Family members can potentially provide general care and psychological support for TB patients, hence treatment management by family members can be an effective strategy to increase treatment success among migrant TB patients in some settings. Retreated patients are more likely than new TB cases to develop MDR TB, which occurred in 21% of retreated TB patients in Shanghai in 2010 [23]. While the current national TB control program in China provides free first-line anti-TB drugs to new and retreated patients, first-line anti-TB drugs may be ineffective in TB cases with drug resistance. If drug-resistant TB patients in China want to be cured, they have to buy the second-line anti-TB drugs out-of-pocket, a cost between $1,979 to $8,196 per patient [24]. Since 2004, the Shanghai government has a policy and commitment to provide the same anti-TB health services to migrants that are available to the local residents. However, additional interventions, including food and housing subsidies, strengthened case management by health care workers and keeping the job position during their treatment course may be needed by urban migrants to improve treatment outcomes. 
